# Patient–ventilator asynchrony during critical care transport: evidence and framework

**DOI:** 10.3389/fmed.2026.1965514

**Published:** 2026-10-16

**Authors:** Yutao Ge, Aiyi Zheng, Peng Deng, Ping Zhang, Guo Tang, Rong Yao

**Affiliations:** Emergency Department, West China Hospital, Sichuan University, Chengdu, Sichuan, China

**Keywords:** critical care transport, mechanical ventilation, patient–ventilator asynchrony, transport ventilator, waveform monitoring

## Abstract

Transport of invasively ventilated patients is a necessary but vulnerable phase of critical care, yet patient–ventilator asynchrony (PVA) has rarely been measured directly during transport. This structured narrative review integrates transport studies, ventilator bench evaluations, ICU physiology, and monitoring research, with a targeted PubMed update on 1 October 2026. Transport-associated waveform reports, clinical labels, and treatment records provide different levels of diagnostic certainty. We identified no prospective cohort continuously comparing PVA before, during, and after transport using synchronized ventilator and patient-effort signals; incomplete historical search documentation limits this absence statement. Transport-specific incidence and clinical consequences remain uncertain. Bench performance is device- and condition-dependent, and ICU studies show heterogeneous outcome associations and intervention effects. We therefore propose an unvalidated framework spanning patient vulnerability, circuit integrity, transport ventilator performance, and environmental and operational disturbance. These domains may influence triggering, flow delivery, cycling, and respiratory rhythm through overlapping mechanisms. A proposed safety-first workflow prioritizes effective oxygenation and ventilation, airway–circuit–device assessment, and contextual interpretation of suspected phenotypes. Motion artifact, true triggering disturbances, and indeterminate patterns must be distinguished; advanced effort measurements primarily support research validation. Initial studies should establish synchronized-signal completeness, interpretable recording time, artifact burden, and inter-rater agreement before testing clinical benefit and balancing harms.

## 1 Introduction

Critically ill patients frequently require intrahospital or interhospital transport for diagnostic procedures, therapeutic interventions, specialist care, or definitive treatment. For patients receiving invasive mechanical ventilation, transport temporarily relocates respiratory support from a controlled intensive care unit (ICU) to a setting with restricted space, reduced physical access, mobile equipment, leaner staffing, and greater exposure to handling and environmental disturbance. Prospective studies and systematic reviews document airway, respiratory, equipment, and monitoring events during transport, although definitions and reported frequencies vary substantially (1–4).

Patient–ventilator asynchrony (PVA) describes a mismatch between neural respiratory activity or ventilatory demand and ventilator assistance during breath triggering, inspiratory flow delivery, or cycling (5). In ICU populations, PVA may increase respiratory effort, discomfort, and breath-to-breath variability, while some phenotypes produce breath stacking or excessive tidal volume. A recent systematic review found associations between PVA and longer durations of mechanical ventilation and ICU stay, whereas pooled analyses did not demonstrate a significant association with mortality; causality remains uncertain (6). These concerns fit within the broader goal of lung- and diaphragm-protective ventilation (7).

Critical care transport may modify patient–ventilator interaction through several simultaneous perturbations. Pain, anxiety, sedation, positioning, secretion movement, gas-exchange deterioration, or altered respiratory mechanics may change respiratory demand. The circuit may be extended, reconnected, pulled, compressed, or exposed to moving condensate. A transport ventilator may differ from the ICU ventilator in trigger algorithms, pressurization, cycling, leak compensation, waveform display, and alarms. Motion, limited access, workload, and, during aeromedical transport, reduced ambient pressure can further influence signal fidelity or delivered support. These mechanisms are plausible, but plausibility is not direct proof that transport causes PVA.

In this review, “PVA during transport” refers descriptively to asynchrony observed or suspected within a transport episode. It is not presented as a distinct phenotype, syndrome, or independently established complication. Transport is treated as a potentially sensitive time window in which familiar PVA mechanisms may emerge, worsen, or become harder to detect and correct. This structured narrative review asks four questions: what is directly known about patient–ventilator interaction during critical care transport; which conclusions are extrapolated from ICU, bench, or adjacent evidence; how transport-sensitive perturbations might map to established PVA mechanisms and phenotypes; and how a safety-first clinical and research framework could be tested. The proposed framework is the authors' synthesis and has not undergone formal consensus development or prospective validation (Figure 1).

**Figure 1:**
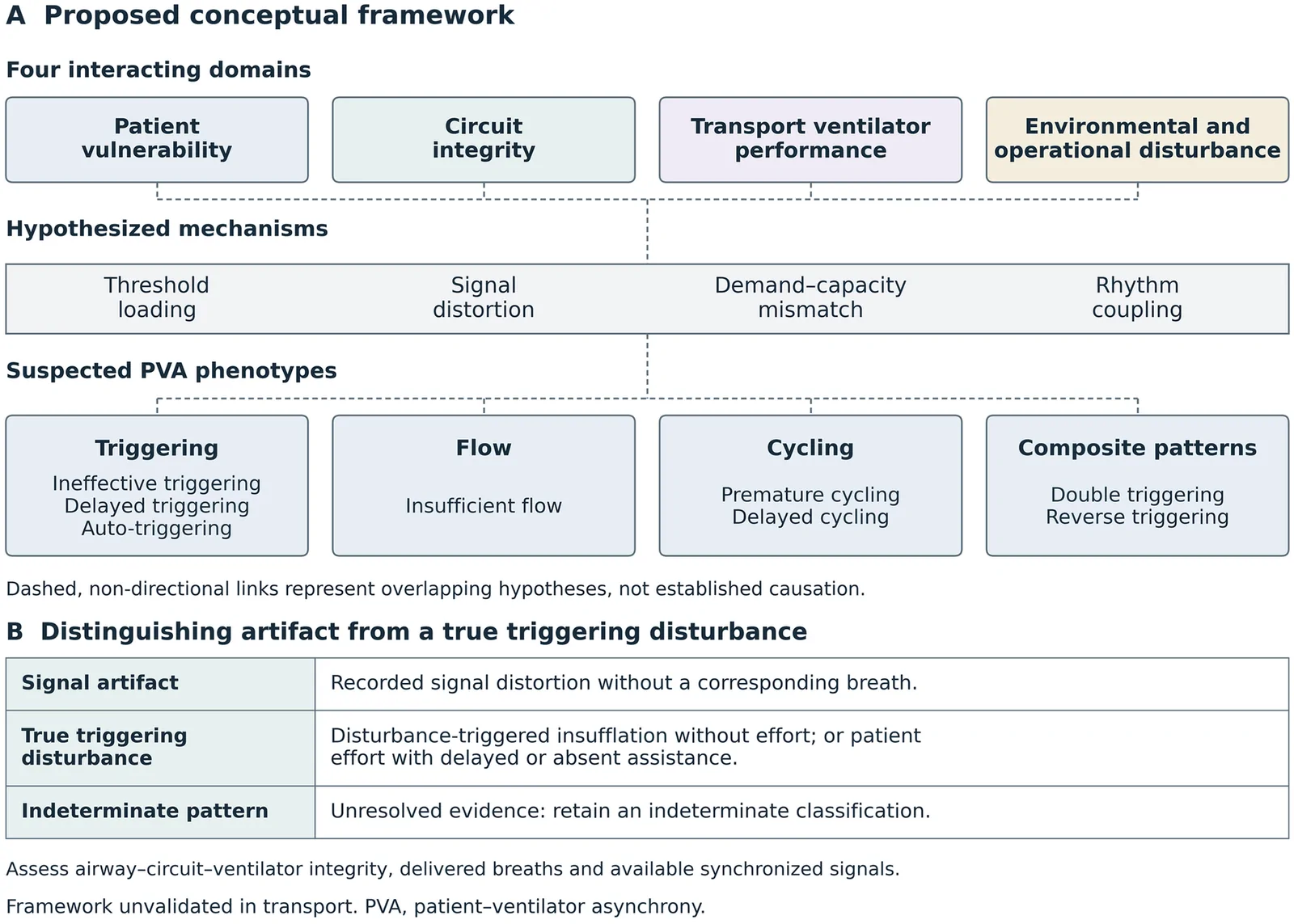
Proposed conceptual framework and interpretation of suspected PVA during critical care transport. **(A)** Four interacting domains may contribute through overlapping hypothesized mechanisms to suspected PVA phenotypes; dashed, non-directional links do not imply one-to-one relationships or established transport-specific causation. **(B)** Examples distinguish recording artifact, true triggering disturbance, and unresolved patterns. These are illustrative distinctions, not stand-alone diagnostic criteria. Assessment integrates airway–circuit–ventilator checks, delivered breaths, the patient's condition, and available synchronized signals; waveforms alone may not establish absent effort. The framework remains unvalidated in transport. PVA, patient–ventilator asynchrony.

## 2 Review approach and scope

This structured narrative review addresses PVA during invasive critical care transport, including intrahospital, interhospital, prehospital, ground, and aeromedical settings. The original review reported searches of PubMed/MEDLINE, Embase, Web of Science Core Collection, Scopus, and the Cochrane Library through 10 July 2026; complete historical queries, language restrictions, and screening responsibilities could not be verified. During revision, four targeted PubMed searches were executed on 1 October 2026 without a language filter, supplemented by citation checking and targeted publisher searches; the executed queries and retrieval yields are provided in Supplementary Material S1. Selection used title/abstract review and targeted full-text checking, with extraction restricted to verifiable information when full text was unavailable. Published reports addressing PVA during actual invasive transport were eligible as direct observations, distinguishing waveform descriptions, clinical labels, and treatment records. Composite transport events, transport safety studies, pediatric NIV, bench experiments, ICU physiology and intervention studies, and monitoring research were eligible as contextual or supporting evidence; unrelated transport or ventilation reports were excluded. Relevant positive, null, and conflicting findings were considered irrespective of whether they supported the framework. Published protocols informed research priorities only. Sources were compared qualitatively by population, transport mode, ascertainment, design, findings, and inferential limits (Table 1). The four domains are an interpretive synthesis, not evidence of validated causal pathways. No formal risk-of-bias grading, independent duplicate screening, or quantitative pooling was performed; the targeted update does not constitute a repeat of all five original database searches.

**Table 1:** Evidence map and limits of inference for PVA during critical care transport.

Evidence source [references]	Population/setting/ascertainment	What the evidence supports	Main limits
Contextual transport safety evidence (1–4)	Intrahospital, interhospital, and out-of-hospital cohorts/reviews; adverse events, alarms, equipment problems, interventions	Establishes respiratory and equipment vulnerability during transport	Not designed to quantify PVA burden; adverse events cannot estimate PVA burden
Guidance and practice evidence (8–13)	Transport safety guidance and ground/aeromedical practice studies; staffing, monitoring, settings, oxygen, sedation	Provides safety processes and describes practice heterogeneity	Does not establish that a workflow or sedation strategy prevents PVA
Monitoring and environmental context (17–19)	Transport EtCO₂–PCO₂ comparison; helicopter cuff-pressure observations; ambulance vibration measurements in neonatal and adult-caregiver mannequins	Identifies safety-monitoring and environmental considerations	Does not identify PVA phenotypes or prove vibration- or altitude-induced PVA
Direct waveform observation (20)	Adults receiving invasive ventilation; 15–20-minute intrahospital transfers; naturalistic pressure, flow, and volume examples	Preliminary visual patterns interpreted as transient PVA	No suitable incidence denominator, synchronized effort reference, standardized adjudication, or causal comparison
Transport-associated case with tracings (21)	Post-arrest emergency transport; vehicle unspecified; accompanying pressure/flow examples interpreted as reverse triggering	Documents an earlier transport-associated observation	EAdi belongs to the separate ICU case; tracing capture location not explicit; no incidence or causal comparison
Direct treatment-based record (11)	460 adults; no neuromuscular blocker during transport. Ground/rotor-wing. Medication for asynchrony: 10/460 (2.2%).	Documents treatment attributed to suspected dyssynchrony	No strict dyssynchrony definition; selected population and narrative ascertainment; treatment rate is not physiological PVA incidence
Direct clinical-event record (24)	36 ventilated COVID-19 patients; fixed-wing Airbus A330 MRTT evacuation; eight PVA/cough events requiring intervention	Documents clinician-recognized events during fixed-wing transport	Events are not necessarily unique patients; no synchronized effort adjudication or pretransport waveform-defined PVA comparator
Direct clinical/treatment record (25)	Fixed-wing C-17/C-130J evacuation series; PVA recorded in five of eight mechanically ventilated patients	Documents suspected PVA and related interventions	Selected pandemic cohort; no standardized waveform adjudication; cannot establish treatment efficacy
Direct treatment-selected record (26)	Ground and helicopter service; 126 adults receiving non-intubation neuromuscular blockade; dyssynchrony indicated in 71 (56.4%)	Describes reasons for neuromuscular blockade during transport	Treatment-selected denominator excludes untreated patients and cannot estimate PVA incidence
Clinical composite record (22)	31 severe-TBI intrahospital transfers; prospective adverse-event collection	24 events in 19 patients; definition includes hardware failure or asynchrony requiring intervention	Composite event rate is not PVA incidence; PVA-specific breakdown not verified from the accessible abstract
Composite treatment record (23)	Rotary-wing interfacility cohort; sedation/paralysis for vital-sign change or dyssynchrony: 15 records (reported 8%)	Documents treatment during transport; no major in-flight adverse events reported	Mixed indications; retrospective ascertainment and selected stabilized patients; cannot isolate PVA
Supporting pediatric NIV evidence (27)	263 bronchiolitis transfers by a pediatric interhospital ground team; retrospective incidents, including leaks and PVA	Shows that suspected PVA is documented in a different transport/ventilation population	14.1% refers to all NIV-related incidents, not PVA alone; different ventilation population and clinical-incident ascertainment
Clinical delivery-method evidence (29)	22 intrahospital transports in 16 patients, randomized by transport; patient-triggered ventilation compared with manual ventilation	Informs stability of respiratory rate, tidal volume, and PEEP	Does not establish an effect on PVA
Transport-device bench evidence (14–16, 28)	Delivery performance under simulated mechanics [14–16]; triggering with simulated effort [15,16]; mild-altitude delivery in a normal-resistance, high-compliance model [28]	Device-dependent performance; several sophisticated or turbine devices performed close to ICU comparators for specified outcomes	Device-, mode-, and condition-specific; neither uniformly poor performance nor clinical equivalence is established
Supporting circuit-load physiology (30)	Mechanical effects of HME use in ventilated patients	Supports physiological interpretation of added resistance and dead space	Application to changing transport circuits is mechanistic extrapolation
Supporting ICU physiology and detection (5, 7, 31–43, 52–57)	Clinical and physiological studies, simulation, reviews, guidance, consensus, and algorithms	Definitions, mechanisms, management principles, and candidate detection methods	Different populations and measurement standards; transport application requires validation
Supporting ICU outcome evidence (6, 44–47)	Cohorts and evidence syntheses; different phenotypes, thresholds, observation periods, and case mixes	Associations with ventilation duration; mortality findings and phenotype associations are inconsistent	Confounding and measurement heterogeneity; association does not establish causation or transport prognosis
Supporting ICU randomized evidence (48–51)	Mode strategies and a bundled PVA-management protocol	Physiological and clinical results differ; some outcomes improve and others do not	Different interventions/comparators; no isolated causal PVA effect or validation of the transport workflow
Published transport protocol (61)	Planned intrahospital AMV trial; primary endpoint is peri-transport PaCO₂ change	Shows an active research direction focused on gas-exchange stability	Protocol only; no effectiveness results or effort-based PVA outcome

Direct denotes observation or documentation during actual invasive transport, not equal diagnostic certainty. Waveform descriptions, clinician labels, composite events, and treatment records are distinct. Contextual and supporting evidence does not validate the proposed transport framework. AMV, adaptive minute ventilation; EAdi, electrical activity of the diaphragm; HME, heat and moisture exchanger; ICU, intensive care unit; MRTT, Multi-Role Tanker Transport; NIV, noninvasive ventilation; PVA, patient–ventilator asynchrony; TBI, traumatic brain injury; PaCO₂, arterial partial pressure of carbon dioxide; PEEP, positive end-expiratory pressure; EtCO₂, end-tidal carbon dioxide; PCO₂, partial pressure of carbon dioxide.

## 3 What is directly known during critical care transport

### 3.1 Transport is a vulnerable interval, but adverse events are not PVA

Most clinical transport evidence concerns safety rather than direct assessment of PVA. Transport cohorts and reviews describe oxygenation or ventilation changes, airway and circuit problems, equipment malfunction, loss of monitoring, and unplanned intervention (1–4). These studies establish that the airway–circuit–ventilator system is exposed to clinically relevant disturbance. These safety outcomes do not provide a measure of physiological PVA burden.

Tachypnea, reduced tidal volume, elevated airway pressure, desaturation, or altered end-tidal carbon dioxide (EtCO₂) may coexist with PVA, but each can also reflect tube displacement, obstruction, circuit disconnection, bronchospasm, loss of positive end-expiratory pressure (PEEP), disease progression, perfusion change, or artifact. Transport adverse-event data should therefore justify vigilance and structured troubleshooting, not be reinterpreted as surrogate incidence data for PVA.

### 3.2 Practice, monitoring, and airway conditions change during transport

Professional guidance emphasizes risk–benefit assessment, trained personnel, appropriate monitoring, oxygen and power reserves, contingency planning, and structured handoff (8–10). Actual practice remains heterogeneous. A ground/rotor-wing transport cohort reported frequent deep sedation (11). An air-medical cohort documented incomplete height documentation for predicted-body-weight–based ventilation (12), while an international helicopter-service survey described heterogeneous ventilation resources and monitoring practices (13). Settings that appear numerically equivalent across devices may not produce identical trigger response, pressurization, cycling, or delivered tidal volume (14–16).

Capnography provides a continuous safety trend during transport, but the difference between EtCO₂ and blood carbon dioxide tension (PCO₂) is variable (17). EtCO₂ cannot identify a PVA phenotype. It should prompt airway, circuit, ventilator, and patient reassessment rather than serve as a PVA diagnostic test. During non-pressurized aeromedical transport, endotracheal tube cuff pressure can increase as altitude rises (18). This supports cuff reassessment; the study did not establish a causal link to leak or waveform-defined PVA. An ambulance experiment using neonatal and adult-caregiver mannequins documented vibration exposure (19), but whether such vibration causes auto-triggering in invasively ventilated adults has not been established.

### 3.3 Direct PVA evidence is sparse and methodologically limited

A 2026 naturalistic report presented pressure, flow, and volume waveform examples before and during 15–20-minute intrahospital transfers in adults receiving invasive ventilation. Transient patterns interpreted as PVA, including double triggering, were described during transport. However, the observational component did not provide a transport denominator suitable for incidence estimation, a standardized adjudication process, a synchronized patient-effort reference, or clinical outcome analysis. It therefore provides preliminary visual evidence, not an estimate of PVA burden or proof that transport caused the observed events (20).

An earlier two-case report described regular gasping during emergency transport after cardiac arrest, with accompanying pressure and flow tracings interpreted as reverse triggering. Synchronized EAdi was obtained in the separate ICU case, not the transport case. The transport mode and exact location of tracing capture were not specified. This selected neurological presentation supports recognition of a transport-associated observation, but does not establish incidence or transport-induced causation (21).

Other transport cohorts used composite outcomes. A prospective study of 31 intrahospital transfers in severe traumatic brain injury defined adverse events as hardware failure or ventilator asynchrony requiring intervention; its 24 events in 19 patients cannot be interpreted as PVA-specific incidence (22). An earlier rotary-wing interfacility cohort grouped sedation or paralysis for vital-sign changes or ventilator dyssynchrony together (15 records; reported as 8%). It recorded no major in-flight adverse events, illustrating that transport outcomes are not uniformly unfavorable, although retrospective ascertainment and selected, stabilized patients limit reassurance (23). Neither composite category isolates physiological PVA.

Retrospective transport series also recorded clinician-identified PVA or related management. In a cohort of 460 adults undergoing ground or rotor-wing critical care transport who did not receive neuromuscular blockers during transport, 10 (2.2%) were documented as receiving medication for ventilator asynchrony (11). During fixed-wing collective evacuation using an Airbus A330 MRTT, a cohort of 36 ventilated patients with COVID-19 acute respiratory distress syndrome had eight events labelled “patient–ventilator asynchrony, including cough” that required intervention (24). In a separate fixed-wing evacuation series using C-17 and C-130J aircraft, clinician-documented PVA management was reported in five of eight mechanically ventilated patients (25). In a selected cohort of 126 adults transported by a ground and helicopter service who received neuromuscular blockade for indications other than intubation, ventilator dyssynchrony was the documented indication in 71 (56.4%); this selected denominator precludes incidence estimation (26). These reports relied on event- or treatment-based ascertainment rather than prespecified waveform adjudication with synchronized effort signals. The Turc and Davis cohorts were selected pandemic evacuation populations, and the Esteves denominator was restricted to patients receiving neuromuscular blockade. Medication use documents a clinician's indication for treatment, not physiological confirmation of PVA or evidence of treatment efficacy. These observations should therefore be interpreted separately from waveform descriptions (20, 21).

As supporting evidence from a different ventilation population, a 2025 pediatric interhospital ground-transport cohort of noninvasive ventilation (NIV) for bronchiolitis reported incidents in 14.1% of 263 transfers; leaks and PVA were among the common minor complications (27). This retrospective clinical-incident report involved pediatric NIV and cannot establish waveform-defined PVA burden in invasively ventilated adults or children.

Together, these reports show that PVA can be observed, recorded, or managed during real transport, but none establishes transport-specific incidence, phenotype distribution, change from a stable baseline, or independent clinical consequences. Within the available sources, including the targeted update through 1 October 2026, we identified no prospective cohort of invasively ventilated patients that synchronized pretransport, in-transit, and post-transport ventilator waveforms with an accepted patient-effort reference. Current evidence therefore cannot determine whether transport increases overall PVA burden, which perturbations are independently responsible, or whether correcting suspected PVA during transport improves outcomes. Table 1 separates transport context, waveform observations, clinical records, and mechanistic extrapolation, with the limits of each evidence source.

### 3.4 Transport ventilator evidence is informative but indirect

Bench evaluations show device-dependent pressurization and tidal-volume delivery under simulated mechanics (14–16), with triggering performance evaluated using simulated efforts (15, 16). Findings are not uniformly unfavorable: several sophisticated transport ventilators achieved triggering performance comparable to ICU-like devices, and tested turbine transport ventilators performed close to an ICU ventilator for many volume-delivery and PEEP conditions (15, 16). At simulated altitudes of 1,500 and 2,500 m, tidal-volume accuracy varied by device; most tested ventilators remained within the study's accuracy limits under normal-resistance, high-compliance lung-model conditions (28). These results depend on device generation, mode, simulated mechanics, and test conditions. They caution against both treating all transport ventilators as inferior and assuming that favorable bench performance establishes clinical equivalence. Simulated effort and bench asynchrony can be quantified, but clinical PVA burden and patient-centered effects during transport require separate evaluation.

In a prospective single-center study, 22 intrahospital transports involving 16 spontaneously breathing patients were randomized by transport to patient-triggered mechanical or manual ventilation. On return to the ICU, manual ventilation was associated with a higher respiratory rate and greater variation in tidal volume and PEEP (29). The reported outcomes concern ventilatory stability and do not establish an effect on PVA.

Circuit components also matter. Heat and moisture exchangers (HMEs) can increase dead space and resistive load in ventilated patients (30). During transport, added connectors, filters, secretions, kinking, leaks, or condensate may further change the effective load or the pressure and flow signal reaching the ventilator. The link from these disturbances to a specific PVA phenotype remains a mechanistic extrapolation unless synchronized effort and ventilator signals are recorded.

### 3.5 Transport settings are not interchangeable

Transport settings share a need for continuity of life support, but their exposures are not equivalent. Intrahospital planning must account for the route, elevators, procedural areas, equipment, staffing, and handover (8, 10). Interhospital ground transfer may involve longer journeys, while movement and the vehicle environment can restrict access to the patient and equipment (8). Prehospital mechanical ventilation requires trained personnel and an individualized ventilation strategy (9). Air transport practice and resources vary across services (12, 13). During unpressurized helicopter transport, cuff pressure may increase with altitude (18). Ground-ambulance vibration has been measured in experiments using neonatal and adult-caregiver mannequins (19). These sources describe distinct settings and exposures; they do not establish their independent effects on PVA.

These differences may modify both the probability of a true patient–ventilator interaction problem and the probability that it will be detected. A brief circuit perturbation may dominate an intrahospital episode, whereas changing respiratory drive, secretion burden, temperature, analgesia-sedation exposure, or cumulative device–patient mismatch may become more important during a prolonged transfer. Reduced ambient pressure is specific to altitude exposure, while motion artifact and restricted visual access vary with vehicle, route, restraint system, and staffing. Consequently, a single pooled label of “transport” may conceal clinically relevant effect modification. Future studies should prespecify transport type, route phases, duration, handling events, staffing, device changes, and environmental exposures, and should compare each in-transit interval with a stable predeparture baseline and a post-arrival recovery period. Until such data exist, conclusions derived from one transport mode should not be assumed to apply to another.

## 4 Proposed transport-sensitive mechanism-to-phenotype framework

The proposed framework combines four interacting domains: patient vulnerability, circuit integrity, transport ventilator performance, and environmental and operational disturbance. These domains describe sources of influence, whereas threshold loading, signal distortion, demand–capacity mismatch, and ventilator–patient rhythm coupling describe possible mechanisms. Their relationships are overlapping rather than one-to-one: a disturbance may act through several pathways, and a pathway may involve several domains. Figure 1 therefore separates domains, pathways, and phenotypes without implying transport-specific causal validation. Table 2 links these proposed pathways to suspected phenotypes, diagnostic limits, and proposed initial responses. Signal artifact and indeterminate patterns require separate assessment and should not automatically be classified as PVA.

**Table 2:** Transport-sensitive disturbances, suspected PVA phenotypes, and diagnostic limits.

Disturbance/proposed mechanism	Suspected phenotype	Suggestive clue	Diagnostic limit	Proposed initial response^a^
Obstruction, secretions, loaded filter/HME, or insufficient expiration Threshold loading	Ineffective or delayed triggering	Expiratory flow persists; apparent unassisted effort	Check mechanics and expiratory flow; effort timing may remain uncertain	Correct obstruction; suction if indicated. Consider expiratory time, PEEP, and trigger settings in context.
Leak, condensate, partial disconnection, or circuit/sensor motion Signal distortion	Auto-triggering; possible ineffective or delayed triggering	Unexpected machine breaths; unstable baseline; onset with handling	Separate distorted recording from actual insufflation; waveforms alone may not establish absent effort	Correct leak/disconnection, drain condensate, secure circuit/sensors; assess mechanical causes before trigger changes.
High drive with limited flow or slow pressurization Demand–capacity mismatch	Insufficient flow	Pressure scooping; distress; persistent inspiratory demand	Waveforms support suspicion; effort signals strengthen attribution	Treat reversible high drive; consider flow, rise time, or support within protective and device limits.
Neural inspiration outlasts assistance Demand–capacity mismatch	Premature cycling; possible double triggering or stacking	Paired breaths with short expiration; excessive combined volume	Paired breaths are visible; an effort signal clarifies whether one prolonged effort spans both	Address drive; consider inspiratory time, flow, and cycling while limiting injurious volume and pressure.
Assistance outlasts neural inspiration Demand–capacity mismatch	Delayed cycling	Late pressure rise or expiratory distortion	Effort offset may not be identifiable from ventilator waveforms alone	Consider cycling threshold, inspiratory time, support, and leak compensation according to mode.
Insufflation precedes inspiratory effort Rhythm coupling	Reverse triggering, with or without stacking	Post-insufflation deflection; regular or irregular pattern	Waveforms support suspicion; synchronized EAdi/Pes strengthens physiological interpretation	Assess stacked volume/pressure; review rate, inspiratory time, mode, and sedation targets in context.
Motion, noise, sensor displacement, or poor signal Comparator: artifact/indeterminate	No definite PVA assignment	Movement-linked trace or disagreement among signals	A disturbance-triggered delivered breath is not recording artifact alone	Check signal quality, delivered breaths, and patient; reassess when stationary if safe; retain uncertainty when unresolved.

a After stabilization. These proposed transport adaptations are not a validated treatment protocol. Effort-reference measurements primarily support research and are not prerequisites for urgent bedside action. EAdi, electrical activity of the diaphragm; HME, heat and moisture exchanger; PEEP, positive end-expiratory pressure; Pes, esophageal pressure; PVA, patient–ventilator asynchrony.

### 4.1 Patient vulnerability and threshold loading

Pain, anxiety, fever, hypoxemia, acidosis, or agitation can increase respiratory drive and effort. If neural inspiratory time exceeds ventilator inspiratory time, or delivered flow or pressurization is inadequate for demand, premature cycling, insufficient flow, or double triggering may occur (5, 31). Conversely, excessive sedation, neurologic impairment, weakness, or fatigue can reduce the signal generated by an inspiratory effort and contribute to delayed or ineffective triggering. Increased resistance, bronchospasm, secretions, expiratory flow limitation, or insufficient expiratory time can produce dynamic hyperinflation and intrinsic PEEP. This threshold load must be overcome before the ventilator trigger is reached (31–33).

A stable predeparture setting may become mismatched after positioning, clinical deterioration, or a device change. Individual responses to altered pressure support are variable (34), and PVA burden varies among mechanically ventilated patients with acute brain injury in the ICU (35). These data identify vulnerabilities rather than transport-specific phenotypes.

### 4.2 Circuit integrity and signal distortion

Handling, extension, reconnection, traction, compression, leaks, and moving condensate can affect both support delivery and the signals used to detect effort. A leak may shift baseline flow or pressure, attenuate true effort, or interact with leak compensation. Mechanical oscillation of the circuit, moving condensate, or movement of pressure and flow sensors may produce fluctuations that resemble respiratory effort. Ambulance vibration has been documented in an experiment using neonatal and adult-caregiver mannequins (19), but its contribution to adult transport PVA remains a mechanistic hypothesis. Three possibilities should be distinguished: distortion of the recorded signal without a corresponding breath; an external disturbance that actually triggers ventilator insufflation without patient effort; and genuine patient effort with delayed or absent assistance. Depending on the device and trigger setting, disturbances may therefore produce auto-triggering, delayed triggering, ineffective triggering, or an indeterminate tracing. Abnormal waveforms should be checked against delivered breaths, other synchronized signals, handling events, and the patient's condition. General PVA guidance supports evaluating reversible mechanical causes, and ventilator-assessment guidance supports systematic patient–device assessment (5, 36). Prioritizing airway and circuit checks before trigger adjustment is the authors' proposed transport adaptation; the sequence itself has not been validated.

### 4.3 Demand–capacity mismatch and cycling

Transport ventilator characteristics determine how effort is translated into assistance. Slow pressurization or limited flow may fail to meet high demand. In obstructive physiology, insensitive triggering or inadequate expiratory time may worsen ineffective efforts. With baseline instability, a highly sensitive trigger may increase spurious breaths. Double triggering is clinically important because paired insufflations can create breath stacking even when the set tidal volume is lung protective. An ICU observational study associated double-triggered and flow-limited breaths with potentially injurious tidal volumes; deeper sedation reduced but did not eliminate dyssynchronous breaths (37). In a nonrandomized ICU physiological study, ventilator adjustment was associated with a greater reduction in severe breath-stacking asynchrony than increased sedation–analgesia (38). These are general physiological principles, not proven comparative effects during transport.

### 4.4 Rhythm coupling and reverse triggering

Reverse triggering denotes inspiratory muscle activity beginning after passive, machine-triggered insufflation; it may show regular entrainment but can also occur irregularly (39–41). It is mainly described during controlled ventilation and may be relevant in deeply sedated or neurologically impaired patients. The transport-associated case described above shows that such patterns have been reported during transfer (21), but does not establish that transport initiates reverse triggering. Changes in ventilator rate, inspiratory time, mode, or sedation during transfer could plausibly alter rhythm coupling. Standard pressure and flow waveforms may identify a characteristic reverse-triggering pattern. Synchronized esophageal pressure (Pes) or electrical activity of the diaphragm (EAdi) can improve diagnostic certainty, resolve mimics, and quantify the phase relationship (42, 43).

### 4.5 Clinical significance and limits of outcome evidence

Clinical interpretation should consider phenotype, duration, clustering, and physiological consequence rather than any isolated abnormal breath. An early ICU cohort associated a high overall asynchrony index with mortality (44), whereas a later cohort associated clusters of ineffective efforts with adverse outcomes but found no outcome association for the overall ineffective-effort index or its >10% threshold (45). A longitudinal COVID-19 ARDS cohort associated double triggering and its clusters with longer ventilation but better ICU survival (46). These observational findings do not establish that PVA causes harm or that double triggering is protective. Severity, respiratory drive, mode, sedation, sampling duration, and survival-related exposure time can affect interpretation; a single threshold should not be treated as a universal prognostic marker.

Evidence syntheses also differ. Kyo and colleagues reported associations between high PVA and longer ventilation and higher mortality, with low certainty of evidence (47). The more recent review by de Bie and colleagues associated PVA with longer ventilation and ICU stay but did not find significant pooled mortality associations (6). Differences in eligible populations, phenotype definitions, observation windows, and confounder adjustment limit direct comparison. Neither synthesis proves that reducing an asynchrony index improves survival, and neither supplies transport-specific effect estimates.

Intervention findings are similarly heterogeneous. In one multicenter ICU trial, neurally adjusted ventilatory assist reduced asynchrony but did not improve the primary 48-hour support outcome or 28-day ventilator-free days; some secondary outcomes favored the intervention (48). Another multicenter trial reported more ventilator-free days without a significant hospital-mortality difference (49). In contrast, the larger PROMIZING trial found no significant acceleration of successful liberation with proportional-assist ventilation compared with pressure support (50). These mode-strategy trials differ in populations and interventions and do not isolate the causal effect of correcting PVA. A small, single-center randomized trial of a bundled PVA-management protocol reported shorter ventilation and ICU stay without a significant mortality difference (51). However, systematic PVA recording was confined to the intervention arm, care delivery was unblinded, and sedation, ventilator adjustment, and weaning were combined. Consequently, it cannot establish that a between-group reduction in physiological PVA mediated the reported benefit. These findings justify testing transport-specific benefit and harm rather than assuming either effectiveness or futility.

## 5 Recognition and staged clinical response

The following approach is proposed by the authors and has not been prospectively evaluated for diagnostic accuracy, safety, or outcome benefit. It should be adapted to local protocols, team competencies, and device capability. It does not replace established emergency airway or ventilation procedures.

### 5.1 Detection should follow a hierarchy

The first task is to detect failure of oxygenation, ventilation, hemodynamics, or system integrity. Clinical examination and available monitoring—including peripheral oxygen saturation (SpO₂), EtCO₂, airway pressure and minute-ventilation alarms, hemodynamics, and comfort—form the proposed safety-alert tier (10, 36). These signals are nonspecific and do not diagnose PVA. Pressure, flow, and volume waveforms form a second tier that can support phenotype suspicion. Visual recognition is experience-dependent and has limited sensitivity (52), while motion and circuit artifact further reduce certainty. In simulated scenarios, displaying an estimated muscle-pressure curve improved identification by healthcare professionals (53); this finding is distinct from clinical validation using a measured effort reference. A 2026 modified Delphi consensus classified ineffective triggering, reverse triggering, double triggering, auto-triggering, insufficient flow, premature cycling, and delayed cycling as clinically relevant PVA. The panel considered waveform-only identification feasible for five categories but unlikely for auto-triggering and delayed cycling (42). This expert judgment supports selective waveform assessment; it is not a transport diagnostic-accuracy study or a substitute for resolving uncertain effort timing. In practice, suspected motion artifact warrants inspection of the airway, circuit, connections, condensate, and sensors, followed by comparison with clinical observations and other available signals. When feasible and safe, reassessment during a stationary interval can help determine whether an abnormal pattern persists; disappearance with movement cessation alone does not establish its physiological cause. Unresolved segments should be documented as indeterminate rather than assigned a definite phenotype.

When technically valid, synchronized Pes and/or EAdi can help characterize mechanical inspiratory effort or diaphragmatic activation, respectively, and their timing relative to ventilator assistance (43, 54). During transport, these measurements are primarily reference methods for validation studies and uncertain mechanistic classification, not prerequisites for routine bedside recognition or urgent treatment. Routine decisions must integrate the patient's condition, systematic airway–circuit–ventilator checks, and available pressure, flow, and volume waveforms. Automated detection can quantify selected phenotypes in ICU datasets (39, 55–57). Screen-recording approaches such as SmartAlert offer a way around some proprietary data interfaces, but internal validation against expert labels does not establish physiological accuracy or transport robustness (55). Motion, leaks, signal loss, device heterogeneity, and scarce annotated transport data remain barriers to routine reliance. Table 3 distinguishes clinical assessment from research confirmation and investigational surveillance; it is not a sequence of mandatory bedside tests.

**Table 3:** Proposed levels of assessment and diagnostic certainty for PVA during transport.

Level and use	Inputs	Key question	Diagnostic status	Scope and limits
1. Safety alert Routine clinical assessment	SpO₂, EtCO₂ value/waveform, respiratory rate, hemodynamics, measured ventilation, and alarms	Is oxygenation, ventilation, circulation, or system integrity deteriorating?	Not diagnostic of PVA	Prompts stabilization and equipment assessment; cannot distinguish PVA from obstruction, disconnection, perfusion change, disease, sedation, or artifact
2. Phenotype suspicion Routine clinical assessment	Synchronized airway pressure, flow, and volume waveforms plus observation	Is there a recognizable interaction pattern?	Can support suspicion of selected phenotypes; waveform-only identification is particularly uncertain for auto-triggering and delayed cycling	Combine available waveforms with patient observation and systematic airway–circuit–ventilator checks. Motion may mimic PVA; unresolved patterns remain indeterminate.
3. Physiological reference Research use	Technically valid EAdi and/or Pes synchronized with ventilator waveforms	Did effort occur, and when relative to assistance?	Research reference for activation/effort timing	Primarily supports validation studies and uncertain mechanistic classification; not a mandatory bedside step and must not delay stabilization.
4. Automated surveillance Investigational use	Validated algorithms using synchronized, quality-controlled signals	What are the continuous burden, clustering, and phenotype distribution?	Investigational in transport	Requires transport calibration, artifact handling, external validation across devices and environments, and clinical-utility testing

Levels distinguish uses and diagnostic certainty; they are not a mandatory sequence of bedside tests. Levels 1–2 support routine clinical assessment; Levels 3–4 primarily support research validation or investigational surveillance. EAdi, electrical activity of the diaphragm; EtCO₂, end-tidal carbon dioxide; Pes, esophageal pressure; PVA, patient–ventilator asynchrony; SpO₂, peripheral oxygen saturation.

### 5.2 Before departure: prevent avoidable instability and establish a baseline

Preparation should integrate PVA risk into established transport safety processes. The team should confirm the indication and route, roles, monitoring, oxygen and battery reserves, backup ventilation, and rescue plans (8–10). A baseline should record mode and settings, measured tidal and minute volume, respiratory rate, SpO₂, EtCO₂, airway pressures, hemodynamics, comfort, sedation, and any pre-existing asynchrony. Without a baseline, an event first noticed in transit cannot confidently be attributed to transport.

The airway and circuit should be inspected for position, cuff pressure, secretions, leaks, condensate, kinking, and unnecessary resistance. Suctioning should be performed when clinically indicated and according to established guidance (58). Whenever feasible, the patient should undergo a monitored trial on the intended transport ventilator with the planned circuit, humidification system, oxygen source, and settings. The aim is to identify gross intolerance, inaccurate delivery, unstable triggering, or alarm problems. This pragmatic check has not been shown to reduce PVA.

### 5.3 During transport: stabilize first, classify second

When SpO₂, EtCO₂, airway pressure, tidal volume, respiratory rate, waveforms, or clinical status deteriorate, the immediate goal is effective oxygenation and ventilation. Teams should not delay rescue while trying to name a phenotype. Movement should be paused when feasible and safe, help requested, and manual or alternative ventilation used if adequate support cannot be promptly confirmed. After immediate support is secured, the authors propose rapid parallel assessment of the patient, airway, circuit, ventilator, oxygen source, and power, prioritized by the clinical presentation. Check tube position and patency, biting, secretions, and cuff or seal; inspect the circuit from patient to ventilator for disconnection, leak, kinking, traction, compression, condensate, and filter or HME obstruction; verify mode, settings, alarms, delivered volume, oxygen, power, and device function; then reassess pneumothorax, bronchospasm, acute pulmonary or circulatory deterioration, pain, agitation, sedation, respiratory drive, acidosis, fever, and mechanics. Additional sedation should not substitute for correction of airway, circuit, or device causes; after these are excluded, pain, anxiety, respiratory drive, and sedation targets should be treated according to clinical indication (38). As a physiological caution, empirical escalation of sedation or neuromuscular blockade may suppress visible effort-related manifestations while leaving leak, condensate, or device malfunction unresolved. Reduced apparent asynchrony after medication should not be taken as evidence that system integrity has been restored; persistent auto-triggering or signal distortion still requires mechanical reassessment.

### 5.4 Phenotype-informed action after safety threats are excluded

The following actions draw on general PVA physiology and management principles (5, 32, 33). Their prioritization after stabilization and equipment checks is the authors' proposed adaptation to transport; comparative benefit in this setting has not been established. Persistent suspected ineffective triggering warrants assessment of expiratory flow, intrinsic PEEP, resistance, weak effort, and trigger sensitivity; observational ICU evidence links ineffective triggering with overassistance and patient–ventilator mismatch (31). Suspected auto-triggering warrants correction of leak, condensate, and circuit instability before considering a less sensitive trigger. With insufficient flow, premature cycling, or double triggering, assessment should address both patient demand and ventilator delivery. After reversible causes of high drive are addressed, changes to flow, rise time, support, inspiratory time, or cycling may be considered according to the ventilation mode, device capability, and lung-protective limits. If effort repeatedly follows mandatory insufflation with breath stacking, delivered volume and pressure require reassessment while rate, inspiratory time, mode, and sedation targets are reviewed. Standard-waveform patterns may support suspicion of reverse triggering; synchronized EAdi or Pes provides greater certainty for research validation or unresolved mechanistic questions (42, 43).

### 5.5 After arrival: reassess, communicate, and learn

The post-transport period is part of the episode. Reassess the airway, cuff, circuit, settings, measured ventilation, waveforms, SpO₂, EtCO₂, comfort, sedation, respiratory effort, and hemodynamics, and compare them with baseline. Document major alarms, physiological changes, suspected phenotype and diagnostic confidence, airway or circuit abnormalities, setting changes, rescue actions, response, and unresolved concerns. In before–after studies, structured transport processes and checklists were associated with improved task completion or guideline adherence (59, 60); improvement was not uniform across safety items, and the checklist study reported a reduction in the proportion of transports with a full oxygen cylinder (59). These process studies do not establish prevention of PVA or an overall causal safety benefit.

## 6 Limitations and research agenda

The targeted update identified additional transport reports and positive, null, and conflicting ICU findings, but this remains a narrative synthesis. Search logs for the new PubMed queries are available; the original five-database histories and screening responsibilities remain unverified. There was no independent duplicate screening, formal risk-of-bias grading, or quantitative pooling, so selection bias and incomplete retrieval remain possible. Absence statements are confined to the sources identified, not proof that relevant studies do not exist. Transport case reports lack denominators and causal comparators; clinical documentation favors conspicuous or treatment-requiring episodes, while treatment-selected cohorts and composite outcomes further distort interpretation. Bench studies isolate mechanisms under controlled conditions, and ICU trials test strategies over longer periods in different clinical environments. Neither directly estimates the effect of a short transport episode. Differences in route, staffing, motion, ambient pressure, device capability, and baseline physiology limit generalizability. Misclassification between signal artifact and true triggering disturbances remains a particular concern.

### 6.1 Why transport-specific PVA is difficult to measure

Continuous transport PVA measurement is technically demanding. Many transport ventilators do not provide accessible raw pressure, flow, and volume signals at a documented sampling rate, and exported files may differ in format, resolution, timestamp behavior, and alarm metadata. Changing from an ICU ventilator to a transport device can therefore create a discontinuity exactly when baseline comparison is most important. Even when separate monitors capture capnography and vital signs, device clocks may drift or record at different intervals. A synchronization error of only a few seconds can misassign a transient waveform change to movement, suction, a circuit intervention, or a ventilator adjustment. Signal dropout, compression, automatic filtering, and undocumented setting changes further complicate breath-level adjudication.

Motion and circuit disturbances also create a classification problem rather than simple missingness. Vibration, condensate, leak, sensor displacement, or baseline drift may mimic patient effort or obscure it. Pressure, flow, and volume waveforms can support recognition of selected patterns, but they do not directly establish effort occurrence and timing; technically valid synchronized Pes or EAdi can improve classification certainty and characterize the timing of mechanical effort or diaphragmatic activation relative to ventilator assistance (43, 54, 57). Esophageal pressure or diaphragm electrical activity provides a physiological effort reference, but additional instrumentation, calibration, signal-quality checks, and secure acquisition may be impractical in routine transport. A credible study therefore requires a common clock, raw synchronized signals, device and intervention metadata, prespecified artifact and indeterminate categories, and paired expert adjudication, with an effort-reference subset for criterion validation. These requirements explain why adverse-event reports cannot be converted into PVA incidence and why feasibility and signal validity should precede outcome trials.

### 6.2 Validation priorities

Initial prospective studies should prespecify a small set of primary feasibility outcomes: completeness of the required synchronized signals; the proportion of total transport time that is interpretable; the proportion affected by artifact or classified as indeterminate; and agreement between independent trained raters. Signal completeness and interpretability should use stated recording-time denominators, and rater agreement should be reported before consensus adjudication. These outcomes should establish whether reliable measurement is possible before clinical-effectiveness testing. Paired descriptive measurement should cover a stable pretransport baseline, the ventilator and circuit transition, movement, and post-transport recovery. Device model and software, mode and settings, circuit components, oxygen source, route phase, movement, altitude, position, analgesia-sedation, procedures, alarms, and interventions should be time-stamped. A subset should include esophageal pressure, diaphragmatic electrical activity, or another effort reference.

Outcomes should separate mechanism from safety. Studies should report phenotype-specific frequency and duration, percentage of asynchronous breaths or time, clustering, ineffective efforts, double-triggering, stacked tidal volume, change from baseline, and an explicit artifact or indeterminate category. Immediate safety outcomes may include loss of effective ventilation, hypoxemia, marked carbon dioxide change, manual ventilation, airway or circuit intervention, transport interruption, and post-arrival instability. Mortality and duration of ventilation are distal and confounded and should initially be exploratory.

Transport ventilation research is progressing: a published noninferiority-trial protocol compares adaptive minute ventilation with the pretransport mode using peri-transport arterial carbon dioxide change as the primary endpoint (61). This protocol provides no effectiveness results and addresses gas-exchange stability rather than synchronized effort-based PVA measurement. Future transport studies should distinguish these complementary objectives.

Device studies should incorporate spontaneous effort, obstructive and restrictive mechanics, leaks, added resistance, condensate, motion, oxygen-source variation, and simulated altitude, followed by clinical validation. Signal filtering and artifact-aware algorithms are candidates for future transport monitors, but filtering may also remove true effort information. Evaluation should retain raw signals, report preprocessing, and compare classifications with expert adjudication and an effort-reference subset. Automated systems require transport-specific calibration, artifact labeling, external validation across devices and transport modes, and assessment of false alarms. The proposed workflow should first be tested for feasibility, adherence, transport delay, workload, inappropriate setting changes, sedation exposure, and rescue events. Comparative effectiveness should be evaluated only after feasibility and safety are established. Table 4 proposes a minimum dataset and staged validation pathway.

**Table 4:** Proposed minimum research dataset and staged validation pathway.

Item	Specification	Purpose
A1. Patient context	Age/body size, indication, respiratory and neurological disease, baseline gas exchange, mode, spontaneous activity, analgesia-sedation, sedation score, and baseline PVA	Risk adjustment and phenotype interpretation
A2. Transport context	Intrahospital/interhospital; ground/rotor-wing/fixed-wing; indication, duration, route phase, position, movement/vibration periods, altitude/cabin pressure, staffing, and procedures	Characterize environmental and operational exposure
A3. Ventilator and circuit	Manufacturer/model/software; mode and settings; trigger/cycling; leak compensation; oxygen source; airway size/depth; cuff pressure; HME/filter/connectors; leak, suction, and condensate events	Evaluate device-, setting-, and circuit-dependent mechanisms
A4. Synchronized continuous signals	Raw pressure, flow, and volume at a documented breath-level sampling rate; alarms and device metadata; EtCO₂ waveform/value, SpO₂, heart rate, and blood pressure on a common clock	Detect phenotypes and relate them to safety events without treating nonspecific signals as diagnostic
A5. Effort-reference subset	EAdi and/or Pes synchronized with ventilator waveforms, with signal quality and missing intervals documented	Criterion confirmation of effort; essential for validating effort timing and resolving uncertain or mimicked patterns
A6. Event, intervention, and outcomes	Departure/arrival, handling, device/circuit changes, suction, oxygen changes, medications, ventilator adjustments, rescue actions; phenotype burden, clustering, stacking, unsafe excursions, interruption, and immediate consequences	Establish temporal relationships and distinguish mechanism from safety outcomes
A7. Annotation and quality control	Prespecified definitions; artifact/indeterminate category; at least two independent trained raters; agreement before consensus adjudication; missingness, synchronization error, and signal loss	Improve reproducibility and prevent forced misclassification
B1. Feasibility	Four primary outcomes: 1. Required-signal completeness 2. Interpretable proportion of total transport time 3. Artifact/indeterminate proportion 4. Inter-rater agreement before adjudication State recording-time denominators and assess usability across route phases.	Establish measurable signal feasibility and reproducible classification before clinical-outcome studies
B2. Validity and external validation	Compare with expert consensus and synchronized EAdi/Pes, then test across institutions, patients, transport modes, devices, circuits, motion, and altitude	Establish criterion validity, calibration, generalizability, and failure modes
B3. Clinical utility	Evaluate a monitored workflow for workload, delay, alarm burden, inappropriate changes, sedation exposure, rescue actions, unsafe ventilation, and transport interruption	Establish benefit and balancing harms before routine implementation

A1–A7 specify the minimum dataset; B1–B3 describe staged validation. EAdi, electrical activity of the diaphragm; HME, heat and moisture exchanger; Pes, esophageal pressure; PVA, patient–ventilator asynchrony; SpO₂, peripheral oxygen saturation; EtCO₂, end-tidal carbon dioxide.

## 7 Conclusions

Transport-associated waveform descriptions and clinical records warrant attention to PVA, but do not establish its incidence, independent consequences, or causation by transport. Favorable as well as unfavorable device findings and heterogeneous ICU outcome and intervention evidence require a cautious, context-specific interpretation.

The authors' proposed framework prioritizes preparation, rapid restoration of effective ventilation, and exclusion of airway, circuit, and device problems before phenotype-informed ventilator or sedation changes. Prospective studies should first establish signal feasibility and classification validity, then determine whether the proposed workflow improves clinically meaningful outcomes.
