# Supplementary material for: Patient–ventilator asynchrony during critical care transport: evidence and framework
Source: Front Med (Lausanne). 2026 Oct 16;13:1965514. doi: 10.3389/fmed.2026.1965514 (PMC13720711; doi:10.3389/fmed.2026.1965514)
Supplement: Supplementary file 1 [file Table1.docx]

Supplementary Material S1

Patient–ventilator asynchrony during critical care transport: evidence and framework

# Search methods

This structured narrative review addresses patient–ventilator asynchrony during invasive critical care transport. The original review reported searches of PubMed/MEDLINE, Embase, Web of Science Core Collection, Scopus and the Cochrane Library through 10 July 2026. Historical database-specific queries and language restrictions were incompletely documented. During revision, four targeted PubMed searches were performed on 1 October 2026 without a language filter, supplemented by citation checking. Table S1 records the exact executed queries; the other databases were not rerun for this update.

## Table S1. Executed PubMed searches (1 October 2026)

| **Search and yield** | **Executed PubMed query** |
| --- | --- |
| Direct transport evidence 74 records | (asynchron*[Title/Abstract] OR dyssynchron*[Title/Abstract] OR "patient ventilator interaction"[Title/Abstract] OR "reverse triggering"[Title/Abstract]) AND (transport*[Title/Abstract] OR transfer*[Title/Abstract] OR prehospital[Title/Abstract] OR aeromedical[Title/Abstract] OR ambulance*[Title/Abstract]) AND (ventilat*[Title/Abstract] OR respirat*[Title/Abstract]) AND ("1900/01/01"[Date - Publication] : "2026/10/01"[Date - Publication]) |
| Transport ventilator performance 136 records | (transport*[Title/Abstract] OR prehospital[Title/Abstract] OR aeromedical[Title/Abstract]) AND ventilator*[Title/Abstract] AND (bench[Title/Abstract] OR "lung model"[Title/Abstract] OR triggering[Title/Abstract] OR synchron*[Title/Abstract] OR randomized[Title/Abstract]) AND ("1900/01/01"[Date - Publication] : "2026/10/01"[Date - Publication]) |
| PVA and clinical outcomes 448 records | ventilat*[Title/Abstract] AND (asynchron*[Title/Abstract] OR dyssynchron*[Title/Abstract]) AND (outcome*[Title/Abstract] OR mortality[Title/Abstract] OR "systematic review"[Title/Abstract] OR randomized[Title/Abstract] OR randomised[Title/Abstract] OR randomly[Title/Abstract]) AND ("1900/01/01"[Date - Publication] : "2026/10/01"[Date - Publication]) |
| Proportional ventilation trials 158 records | ("proportional assist ventilation"[Title/Abstract] OR "proportional-assist ventilation"[Title/Abstract] OR "neurally adjusted ventilatory assist"[Title/Abstract]) AND (randomized[Title/Abstract] OR randomised[Title/Abstract] OR randomly[Title/Abstract] OR "Randomized Controlled Trial"[Publication Type]) AND (adult*[Title/Abstract] OR patient*[Title/Abstract]) AND ("1900/01/01"[Date - Publication] : "2026/10/01"[Date - Publication]) |

The searches returned 816 records before deduplication and 737 unique records after deduplication by PMID: 735 journal records and two book chapters. The book chapters were not used as study evidence. Retrieval counts are not counts of included studies.

## Study selection and synthesis

Relevant sources were selected through title and abstract review and targeted full-text checking. Reports of asynchrony during actual invasive transport were classified as direct evidence, distinguishing waveform observations, clinical labels and treatment records. Transport safety, pediatric noninvasive ventilation, bench experiments, ICU physiology and interventions, and monitoring studies were classified as contextual or supporting evidence. Unrelated reports were excluded. Positive, null and conflicting findings were compared qualitatively by population, transport mode, ascertainment and study limitations. No independent duplicate screening, formal risk-of-bias grading or quantitative pooling was performed.

# Expanded search terms

Table S2 provides candidate terms for further searching. These expanded searches have not been executed, and the retrieval counts in Table S1 do not apply to them.

## Table S2. Search concepts and expanded terms

| **Keyword or search concept** | **Corresponding search expression** |
| --- | --- |
| PVA and patient–ventilator interaction | ("Patient-Ventilator Asynchrony"[MeSH Terms]) OR (asynchron*[Title/Abstract]) OR (dyssynchron*[Title/Abstract]) OR ("patient-ventilator asynchrony"[Title/Abstract]) OR ("patient ventilator asynchrony"[Title/Abstract]) OR ("patient-ventilator dyssynchrony"[Title/Abstract]) OR ("patient-ventilator interaction*"[Title/Abstract]) OR ("patient ventilator interaction*"[Title/Abstract]) OR ("patient-ventilator synchron*"[Title/Abstract]) OR ("patient-ventilator mismatch"[Title/Abstract]) OR ("patient-ventilator discordance"[Title/Abstract]) |
| Mechanical ventilation and respiratory support | ("Respiration, Artificial"[MeSH Terms]) OR (ventilat*[Title/Abstract]) OR ("mechanical ventilation"[Title/Abstract]) OR ("mechanically ventilated"[Title/Abstract]) OR ("invasive mechanical ventilation"[Title/Abstract]) OR ("invasive ventilation"[Title/Abstract]) OR ("artificial ventilation"[Title/Abstract]) OR ("artificial respiration"[Title/Abstract]) OR ("positive-pressure ventilation"[Title/Abstract]) OR ("positive pressure ventilation"[Title/Abstract]) OR ("assisted ventilation"[Title/Abstract]) OR ("ventilatory support"[Title/Abstract]) |
| Transport settings and prehospital care | ("Transportation of Patients"[MeSH Terms]) OR (transport*[Title/Abstract]) OR (transfer*[Title/Abstract]) OR ("patient transport*"[Title/Abstract]) OR ("patient transfer*"[Title/Abstract]) OR ("critical care transport*"[Title/Abstract]) OR ("intensive care transport*"[Title/Abstract]) OR (intrahospital[Title/Abstract]) OR ("intra-hospital"[Title/Abstract]) OR (interhospital[Title/Abstract]) OR ("inter-hospital"[Title/Abstract]) OR (interfacility[Title/Abstract]) OR ("inter-facility"[Title/Abstract]) OR (prehospital[Title/Abstract]) OR ("pre-hospital"[Title/Abstract]) OR ("out-of-hospital"[Title/Abstract]) OR ("critical care retrieval"[Title/Abstract]) OR ("medical retrieval"[Title/Abstract]) OR ("retrieval medicine"[Title/Abstract]) |
| Ground and aeromedical transport | ("Air Ambulances"[MeSH Terms]) OR ("ground transport*"[Title/Abstract]) OR ("road transport*"[Title/Abstract]) OR (ambulance*[Title/Abstract]) OR ("air transport*"[Title/Abstract]) OR ("air medical transport*"[Title/Abstract]) OR (aeromedical[Title/Abstract]) OR ("aero-medical"[Title/Abstract]) OR ("aeromedical evacuation"[Title/Abstract]) OR ("medical evacuation"[Title/Abstract]) OR ("air ambulance*"[Title/Abstract]) OR (helicopter*[Title/Abstract]) OR ("helicopter emergency medical services"[Title/Abstract]) OR ("rotor-wing"[Title/Abstract]) OR ("rotor wing"[Title/Abstract]) OR ("rotary-wing"[Title/Abstract]) OR ("rotary wing"[Title/Abstract]) OR ("fixed-wing"[Title/Abstract]) OR ("fixed wing"[Title/Abstract]) OR ("in-flight"[Title/Abstract]) OR (inflight[Title/Abstract]) |
| Transport ventilators and device types | ("Ventilators, Mechanical"[MeSH Terms]) OR (ventilator*[Title/Abstract]) OR ("transport ventilator*"[Title/Abstract]) OR ("portable ventilator*"[Title/Abstract]) OR ("emergency ventilator*"[Title/Abstract]) OR ("prehospital ventilator*"[Title/Abstract]) OR ("turbine-driven ventilator*"[Title/Abstract]) OR ("turbine ventilator*"[Title/Abstract]) OR ("pneumatic ventilator*"[Title/Abstract]) OR ("gas-powered ventilator*"[Title/Abstract]) OR ("ICU ventilator*"[Title/Abstract]) OR ("intensive care ventilator*"[Title/Abstract]) |
| Ineffective and delayed triggering | ("ineffective triggering"[Title/Abstract]) OR ("ineffective effort*"[Title/Abstract]) OR ("ineffective inspiratory effort*"[Title/Abstract]) OR ("missed triggering"[Title/Abstract]) OR ("missed trigger*"[Title/Abstract]) OR ("wasted effort*"[Title/Abstract]) OR ("delayed triggering"[Title/Abstract]) OR ("trigger delay"[Title/Abstract]) OR ("triggering delay"[Title/Abstract]) OR ("trigger failure"[Title/Abstract]) |
| Auto-triggering | ("auto-triggering"[Title/Abstract]) OR (autotriggering[Title/Abstract]) OR ("auto triggering"[Title/Abstract]) OR ("auto-triggered breath*"[Title/Abstract]) OR ("autotriggered breath*"[Title/Abstract]) OR ("false triggering"[Title/Abstract]) |
| Flow asynchrony and flow-demand mismatch | ("flow asynchrony"[Title/Abstract]) OR ("flow dyssynchrony"[Title/Abstract]) OR ("flow starvation"[Title/Abstract]) OR ("insufficient flow"[Title/Abstract]) OR ("insufficient inspiratory flow"[Title/Abstract]) OR ("inadequate inspiratory flow"[Title/Abstract]) OR ("flow demand mismatch"[Title/Abstract]) OR ("inspiratory flow mismatch"[Title/Abstract]) |
| Cycling asynchrony | ("cycling asynchrony"[Title/Abstract]) OR ("cycling dyssynchrony"[Title/Abstract]) OR ("premature cycling"[Title/Abstract]) OR ("early cycling"[Title/Abstract]) OR ("delayed cycling"[Title/Abstract]) OR ("late cycling"[Title/Abstract]) OR ("premature cycling-off"[Title/Abstract]) OR ("delayed cycling-off"[Title/Abstract]) OR ("cycling-off criterion"[Title/Abstract]) OR ("expiratory trigger"[Title/Abstract]) |
| Double triggering and breath stacking | ("double triggering"[Title/Abstract]) OR ("double-triggering"[Title/Abstract]) OR ("double cycling"[Title/Abstract]) OR ("double-cycling"[Title/Abstract]) OR ("breath stacking"[Title/Abstract]) OR ("breath-stacking"[Title/Abstract]) OR ("stacked breath*"[Title/Abstract]) OR ("tidal volume stacking"[Title/Abstract]) |
| Reverse triggering and related entrainment | ("reverse triggering"[Title/Abstract]) OR ("reverse-triggering"[Title/Abstract]) OR ("reverse triggered"[Title/Abstract]) OR ("reverse-triggered"[Title/Abstract]) OR ("respiratory entrainment"[Title/Abstract]) OR ("neural entrainment"[Title/Abstract]) OR ("neuromechanical coupling"[Title/Abstract]) |
| Respiratory drive, loading and assistance | ("respiratory drive"[Title/Abstract]) OR ("inspiratory effort"[Title/Abstract]) OR ("work of breathing"[Title/Abstract]) OR ("neural inspiratory time"[Title/Abstract]) OR ("intrinsic PEEP"[Title/Abstract]) OR ("auto-PEEP"[Title/Abstract]) OR ("auto PEEP"[Title/Abstract]) OR ("dynamic hyperinflation"[Title/Abstract]) OR ("expiratory flow limitation"[Title/Abstract]) OR ("respiratory muscle weakness"[Title/Abstract]) OR (overassistance[Title/Abstract]) OR ("over-assistance"[Title/Abstract]) OR (underassistance[Title/Abstract]) OR ("under-assistance"[Title/Abstract]) |
| Circuit integrity and airway load | ("breathing circuit"[Title/Abstract]) OR ("ventilator circuit"[Title/Abstract]) OR ("circuit leak*"[Title/Abstract]) OR ("air leak*"[Title/Abstract]) OR ("cuff leak*"[Title/Abstract]) OR ("cuff pressure"[Title/Abstract]) OR ("endotracheal tube cuff"[Title/Abstract]) OR ("circuit disconnection"[Title/Abstract]) OR ("circuit traction"[Title/Abstract]) OR ("circuit compression"[Title/Abstract]) OR (kinking[Title/Abstract]) OR (condensate[Title/Abstract]) OR (rainout[Title/Abstract]) OR ("heat and moisture exchanger*"[Title/Abstract]) OR ("heat-moisture exchanger*"[Title/Abstract]) OR ("respiratory filter*"[Title/Abstract]) OR ("filter resistance"[Title/Abstract]) OR ("airway resistance"[Title/Abstract]) OR ("dead space"[Title/Abstract]) OR (secretion*[Title/Abstract]) OR ("airway obstruction"[Title/Abstract]) OR ("endotracheal suction*"[Title/Abstract]) OR ("closed suction*"[Title/Abstract]) |
| Bench models and device performance | ("bench stud*"[Title/Abstract]) OR ("bench test*"[Title/Abstract]) OR ("bench evaluation"[Title/Abstract]) OR ("lung simulator*"[Title/Abstract]) OR ("lung model*"[Title/Abstract]) OR ("test lung"[Title/Abstract]) OR ("breathing simulator*"[Title/Abstract]) OR ("patient simulator*"[Title/Abstract]) OR (mannequin*[Title/Abstract]) OR (manikin*[Title/Abstract]) OR ("ventilator performance"[Title/Abstract]) OR ("triggering performance"[Title/Abstract]) OR ("trigger sensitivity"[Title/Abstract]) OR (pressurization[Title/Abstract]) OR (pressurisation[Title/Abstract]) OR ("tidal volume delivery"[Title/Abstract]) OR ("tidal volume accuracy"[Title/Abstract]) OR ("leak compensation"[Title/Abstract]) OR ("expiratory valve"[Title/Abstract]) |
| Vibration, motion and signal artifacts | (vibration[Title/Abstract]) OR ("vehicle vibration"[Title/Abstract]) OR ("transport vibration"[Title/Abstract]) OR ("whole-body vibration"[Title/Abstract]) OR ("motion artifact*"[Title/Abstract]) OR ("motion artefact*"[Title/Abstract]) OR ("movement artifact*"[Title/Abstract]) OR ("movement artefact*"[Title/Abstract]) OR ("vibration artifact*"[Title/Abstract]) OR ("vibration artefact*"[Title/Abstract]) OR ("sensor noise"[Title/Abstract]) OR ("signal distortion"[Title/Abstract]) OR ("baseline drift"[Title/Abstract]) OR ("flow sensor*"[Title/Abstract]) OR ("pressure sensor*"[Title/Abstract]) OR ("flow transducer*"[Title/Abstract]) OR ("pressure transducer*"[Title/Abstract]) OR ("signal filtering"[Title/Abstract]) |
| Aeromedical environment and pressure changes | (altitude[Title/Abstract]) OR ("cabin altitude"[Title/Abstract]) OR ("cabin pressure"[Title/Abstract]) OR ("ambient pressure"[Title/Abstract]) OR ("barometric pressure"[Title/Abstract]) OR (hypobaric[Title/Abstract]) OR (hypobaria[Title/Abstract]) OR (decompression[Title/Abstract]) OR ("pressurized cabin"[Title/Abstract]) OR ("pressurised cabin"[Title/Abstract]) OR ("altitude compensation"[Title/Abstract]) OR ("hypobaric chamber"[Title/Abstract]) OR ("cuff expansion"[Title/Abstract]) OR ("oxygen supply"[Title/Abstract]) OR ("oxygen consumption"[Title/Abstract]) |
| Operational disturbances and transport resources | ("ventilator change*"[Title/Abstract]) OR ("ventilator switching"[Title/Abstract]) OR ("ventilator transition"[Title/Abstract]) OR ("circuit change*"[Title/Abstract]) OR ("bed transfer"[Title/Abstract]) OR ("stretcher transfer"[Title/Abstract]) OR ("patient handling"[Title/Abstract]) OR (repositioning[Title/Abstract]) OR ("patient positioning"[Title/Abstract]) OR ("interruption of ventilation"[Title/Abstract]) OR ("oxygen depletion"[Title/Abstract]) OR ("battery depletion"[Title/Abstract]) OR ("power failure"[Title/Abstract]) OR ("equipment malfunction"[Title/Abstract]) OR ("transport checklist"[Title/Abstract]) OR ("pretransport checklist"[Title/Abstract]) OR ("pre-transport checklist"[Title/Abstract]) OR (handover[Title/Abstract]) OR (handoff[Title/Abstract]) |
| Waveform assessment and general monitoring | ("ventilator waveform*"[Title/Abstract]) OR ("pressure waveform*"[Title/Abstract]) OR ("flow waveform*"[Title/Abstract]) OR ("waveform analysis"[Title/Abstract]) OR ("ventilator graphic*"[Title/Abstract]) OR ("asynchrony index"[Title/Abstract]) OR ("ineffective triggering index"[Title/Abstract]) OR ("asynchrony burden"[Title/Abstract]) OR ("asynchrony cluster*"[Title/Abstract]) OR (capnograph*[Title/Abstract]) OR ("end-tidal carbon dioxide"[Title/Abstract]) OR (EtCO2[Title/Abstract]) OR ("pulse oximetry"[Title/Abstract]) |
| Effort and neural-signal references | ("esophageal pressure"[Title/Abstract]) OR ("oesophageal pressure"[Title/Abstract]) OR ("esophageal manometry"[Title/Abstract]) OR ("oesophageal manometry"[Title/Abstract]) OR ("electrical activity of the diaphragm"[Title/Abstract]) OR ("diaphragm electrical activity"[Title/Abstract]) OR ("diaphragmatic electrical activity"[Title/Abstract]) OR (EAdi[Title/Abstract]) OR ("inspiratory muscle pressure"[Title/Abstract]) OR ("estimated muscle pressure"[Title/Abstract]) OR (Pmus[Title/Abstract]) OR ("diaphragmatic electromyography"[Title/Abstract]) |
| Automated detection and signal processing | ("asynchrony detection"[Title/Abstract]) OR ("dyssynchrony detection"[Title/Abstract]) OR ("automated detection"[Title/Abstract]) OR ("automatic detection"[Title/Abstract]) OR ("machine learning"[Title/Abstract]) OR ("deep learning"[Title/Abstract]) OR ("neural network*"[Title/Abstract]) OR ("signal processing"[Title/Abstract]) OR ("signal quality"[Title/Abstract]) OR ("artifact rejection"[Title/Abstract]) OR ("artefact rejection"[Title/Abstract]) OR ("waveform classification"[Title/Abstract]) OR ("screen recording"[Title/Abstract]) |
| Ventilation modes and setting adjustments | ("ventilator adjustment*"[Title/Abstract]) OR ("ventilator setting*"[Title/Abstract]) OR ("trigger sensitivity"[Title/Abstract]) OR ("rise time"[Title/Abstract]) OR ("inspiratory time"[Title/Abstract]) OR ("cycling threshold"[Title/Abstract]) OR ("pressure support ventilation"[Title/Abstract]) OR ("proportional assist ventilation"[Title/Abstract]) OR ("proportional-assist ventilation"[Title/Abstract]) OR ("load-adjustable gain factors"[Title/Abstract]) OR ("PAV+"[Title/Abstract]) OR ("neurally adjusted ventilatory assist"[Title/Abstract]) OR (NAVA[Title/Abstract]) OR ("adaptive minute ventilation"[Title/Abstract]) OR ("adaptive support ventilation"[Title/Abstract]) |
| Sedation, analgesia and neuromuscular blockade | (sedation[Title/Abstract]) OR (sedative*[Title/Abstract]) OR (analgesia[Title/Abstract]) OR (analgosedation[Title/Abstract]) OR ("deep sedation"[Title/Abstract]) OR ("sedation protocol*"[Title/Abstract]) OR ("sedation target*"[Title/Abstract]) OR ("neuromuscular blockade"[Title/Abstract]) OR ("neuromuscular blocking agent*"[Title/Abstract]) OR ("muscle relaxant*"[Title/Abstract]) |
| Clinical outcomes and safety | ("clinical outcome*"[Title/Abstract]) OR ("adverse event*"[Title/Abstract]) OR ("respiratory event*"[Title/Abstract]) OR ("treatment intervention*"[Title/Abstract]) OR (mortality[Title/Abstract]) OR ("duration of mechanical ventilation"[Title/Abstract]) OR ("ventilator-free day*"[Title/Abstract]) OR ("ventilator liberation"[Title/Abstract]) OR (weaning[Title/Abstract]) OR ("length of stay"[Title/Abstract]) OR ("patient comfort"[Title/Abstract]) OR ("ventilator-induced lung injury"[Title/Abstract]) OR ("diaphragm dysfunction"[Title/Abstract]) |
| Specific populations and supporting evidence | (adult*[Title/Abstract]) OR ("critically ill"[Title/Abstract]) OR ("critical illness"[Title/Abstract]) OR ("intensive care"[Title/Abstract]) OR (COPD[Title/Abstract]) OR ("chronic obstructive pulmonary disease"[Title/Abstract]) OR (ARDS[Title/Abstract]) OR ("acute respiratory distress syndrome"[Title/Abstract]) OR ("acute brain injur*"[Title/Abstract]) OR ("traumatic brain injur*"[Title/Abstract]) OR (pediatric*[Title/Abstract]) OR (paediatric*[Title/Abstract]) OR (neonat*[Title/Abstract]) OR (infant*[Title/Abstract]) OR ("noninvasive ventilation"[Title/Abstract]) OR ("non-invasive ventilation"[Title/Abstract]) OR (NIV[Title/Abstract]) |

Synonyms and related terms within each row are joined with OR. Enclose each complete row in parentheses before combining concepts with AND. The core search combines asynchrony, including specific phenotypes, AND mechanical ventilation AND transport. Searches of transport ventilation practice and safety may combine mechanical ventilation AND transport without requiring asynchrony terms. Device, mechanism, monitoring and intervention concepts support separate supplementary searches; do not AND every row. * denotes truncation. These expressions use PubMed syntax and require adaptation for other databases.

Breath stacking, entrainment, motion artifacts and general monitoring measures are not all equivalent to physiological asynchrony. Full queries, dates and retrieval counts should be recorded if the expanded searches are executed.
